# Genetic variation in the effect of monoamines on female mating receptivity and oviposition in the adzuki bean beetle, *Callosobruchus chinensis* (Coleoptera: Bruchidae)

**DOI:** 10.1186/s12862-014-0172-5

**Published:** 2014-08-06

**Authors:** Takashi Yamane

**Affiliations:** 1 Present address: Noguchi 350-17, Kakogawa, Japan

**Keywords:** Female mate choice, Seminal fluids, Mating receptivity, Oviposition, Biogenic amines, Adzuki bean beetle

## Abstract

**Background:**

Female mate choice after mating is a strong force in sexual selection and could lead to coevolution of mating traits between the sexes. How females of different genotypes respond to substances in the male ejaculate should be mediated by females’ mate choices. Monoamines regulate animal physiology and behavior, including the post-mating behavior of females of the adzuki bean beetle, *Callosobruchus chinensis* (Coleoptera: Bruchidae). This study examined differences in females’ response to four monoamines (dopamine, octopamine, tyramine, serotonin) between strains from different populations of *C. chinensis*.

**Results:**

Injection with either octopamine or tyramine, two kinds of monoamines significantly reduced female receptivity in two strains with low remating frequencies. None of the four monoamines reduced female receptivity in one strain with high remating frequencies. However, all monoamines reduced it in another strain with high remating frequencies. Oviposition was activated by tyramine on days 1–5 or by serotonin on days 4 and 5 in the two strains with low remating frequencies, but only on day 1 or day 4 in the strains with high remating frequencies.

**Conclusion:**

These differences in female response to monoamines, especially tyramine and serotonin, correspond with results of previous studies. They indicate differences in female response to male substances that reduce receptivity and activate oviposition. These findings suggest relationships between the differences in female response to male substances among populations and mutations in the pathways of monoamine biosynthesis or transmission, which in turn determine female mate choice in response to male substances.

## Background

The lifetime number of matings and fecundity of females differ among animal species [1]–[3]. Males can improve their reproductive success by preventing multiple matings by females, increasing the number of offspring produced, and hastening female oviposition, as a result of reduced sperm competition with other males [4]. However, females can improve their reproductive success by mating with multiple males, leading to increased offspring viability [5], despite the costs of mating [6],[7]. Furthermore, although male ejaculate has a cost [8], eggs are much more valuable to females than sperm are to males [9], so the quality of mates and the effect on offspring quality would be more important for females than for males; thus, females are thought to select males or their sperm so they can adjust the number of offspring sired by a particular males or overall [2],[10].

On this basis, the optimal number of matings and offspring per mating for females might differ from those for males, resulting in sexual conflict over mating traits [11], and cause antagonistic adaptations and counter-adaptations between male and female traits [12],[13]. However, male reproductive traits that impose costs for female fitness can be selected by female mate choice, leading to coevolution between both sexes, if the benefits to females exceed the costs. Because of the importance of similarity and diversity for the genetics of a species, much research has been devoted to antagonistic adaptations or counter-adaptations via sexual conflict and to female mate choice and coevolution of the sexes in terms of traits related to the mating rate and the number of offspring [10],[14]–[19].

Mating can change female behaviors, such as activating oviposition and reducing mating receptivity [4],[10]. Such changes are often caused by substances in the male seminal fluids [20]–[22] that are shown to increase the reproductive success of the males by manipulating females [4]. However, female fecundity and remating rates can differ among populations of a species, possibly reflecting genotypic differences that could be explained by sexual selection via female mate choice based on genotypic differences in the quality of male ejaculate [23]–[26]. However, no studies have defined the physiological components of seminal substances that reflect these variations and cause differences among female genotypes that would lead to sexual selection via female mate choice.

Monoamines are neuroactive substances that affect behavioral and physiological traits in both vertebrates and invertebrates, and they act as neurotransmitters and endocrine disruptors in the central and peripheral nervous systems [27]–[29]. They have several known roles in determining the mating status and behavior of invertebrates: in *Drosophila melanogaster*, octopamine and tyramine regulate egg laying [30]–[32]; in *Caenorhabditis elegans*, serotonin (5-HT) regulates egg laying [33]; in female *Locusta migratoria*, octopamine [34] and tyramine [35],[36] modulate muscle contraction in the spermatheca and oviduct, and serotonin [37] modulates it in the spermatheca, suggesting roles in egg laying; and in Lepidoptera, octopamine and analogs increase egg laying [38], serotonin or its metabolites induce mate-rejection behavior [39], and tyramine reduces the production of sex pheromones [40] within females.

In the adzuki bean beetle, *Callosobruchus chinensis*, male-derived substances reduce female receptivity to mating and activate female oviposition, but the responses of females to these substances and the effects of males differ among strains from different geographic populations [41],[42]. The effects of four artificially injected monoamines (dopamine, octopamine, tyramine, and serotonin) on female receptivity and oviposition in one strain of *C. chinensis* (jC-S) suggested a relationship between these monoamines and female post-mating behavior [43]. Here, I built on this research by comparing the effects of monoamines on female mating receptivity and oviposition among four *C. chinensis* strains derived from populations from different geographic locations, adding three new strains of *C. chinensis* (isC, akC02, and mC; Additional file 1: Table S1) to examine any differences between strains in response to monoamines.

## Results

### Female receptivity

**Figure 1:**
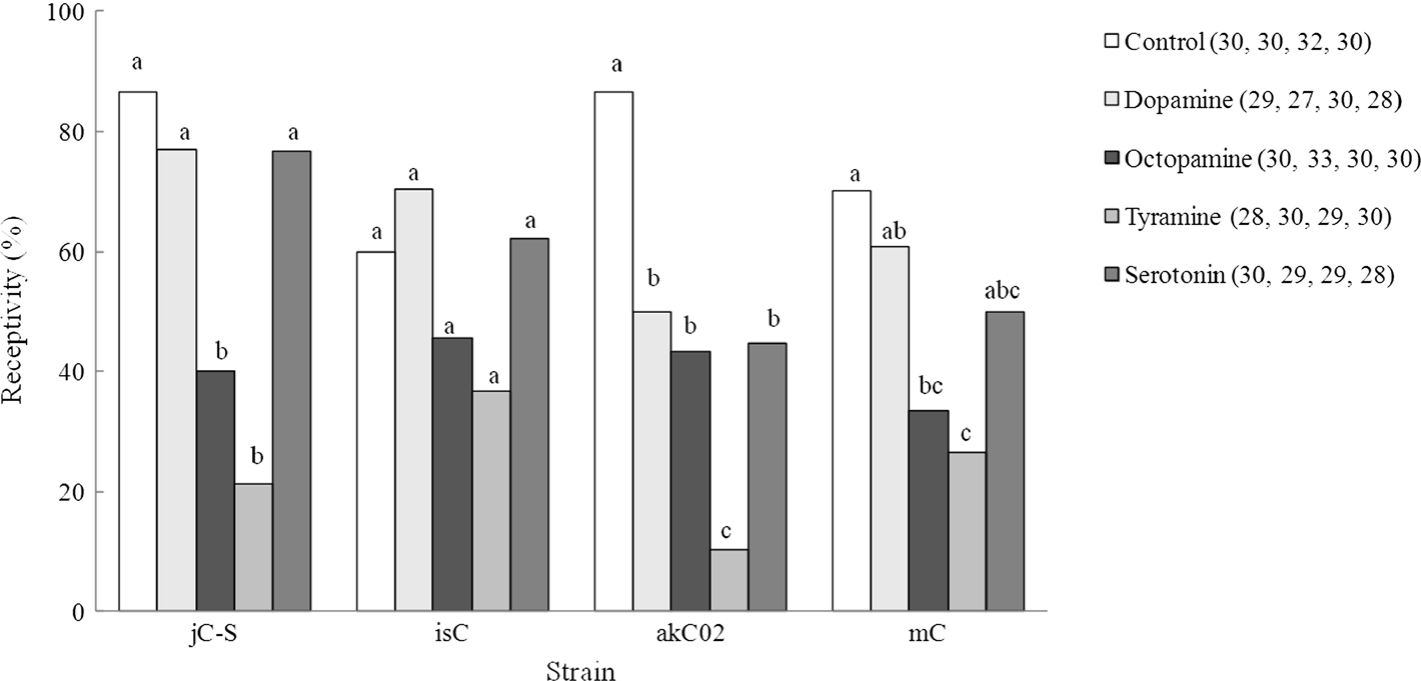
**Effects of monoamines on female mating receptivity.** Receptivity of *Callosobruchus chinensis* females of strains jC-S, isC, akC02, and mC to mating 3–5 h after injection with dopamine, octopamine, tyramine, serotonin, or Milli-Q water (control). Bars labeled with the same letters do not differ significantly within a given strain by the method of Benjamini and Hochberg [53] with a false discovery rate at *P* < 0.05. The numbers in parentheses show the sample size (from left to right, jC-S, isC, akC02, and mC).

Generalized linear model (GLM) results showed significant effects of monoamine treatment on female receptivity and on the interaction between female strain and monoamine treatment, but not of female strain (Table 1). A separate GLM analysis revealed a significant effect of monoamine treatment on female receptivity for each strain except isC (jC-S, likelihood ratio *χ*^*2*^ = 39.510, *P* < 0.0001; isC, *χ*^*2*^ = 8.826, *P* = 0.0656; akC02, *χ*^*2*^ = 38.923, *P* < 0.0001; and mC, *χ*^*2*^ = 16.178, *P* = 0.0028). In jC-S, octopamine and tyramine significantly reduced female receptivity compared with the control, dopamine, and serotonin (Figure 1). In isC, no differences were significant. In akC02, all monoamines significantly reduced receptivity compared with the control, and tyramine reduced it compared with dopamine, octopamine, and serotonin. In mC, octopamine and tyramine reduced receptivity compared with the control, and tyramine reduced it compared with dopamine. In addition, there was difference between strain in receptivity of control (*χ*^*2*^ = 8.565, *P* = 0.0357), but not any between two strains.

**Table 1:** Results of a generalized linear model testing the effects of strain and monoamine treatment on female receptivity

**Deviance**	**Source**	***df***	**Likelihood ratio** ***χ***^**2**^	***P***
705.645	−	*570*	−	−
**-**	Treatment	4	86.270	< 0.0001
**-**	Strain	3	7.711	0.0524
**-**	Strain × treatment	12	21.295	0.0462

### Number of eggs laid by females

**Figure 2:**
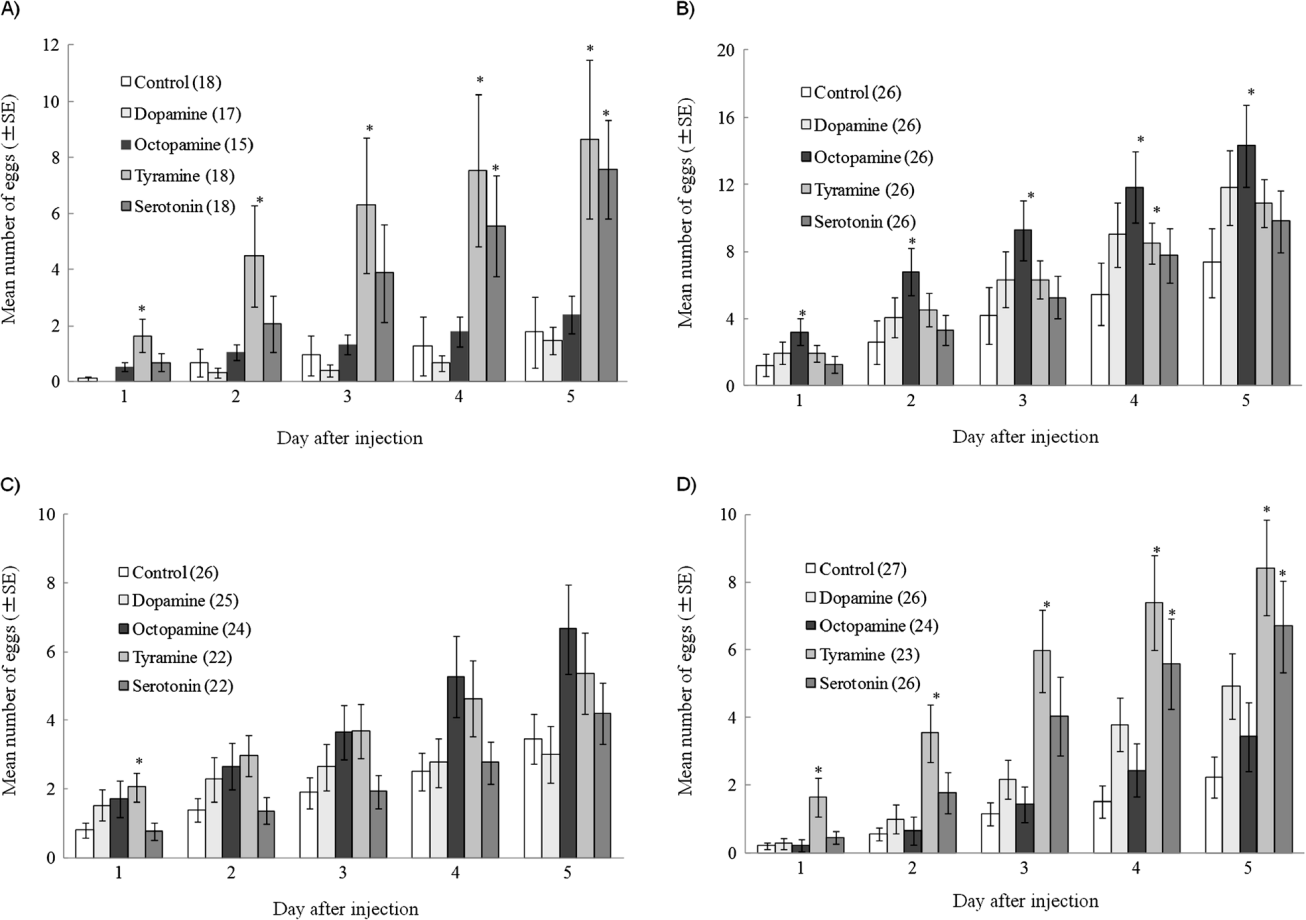
**Effects of monoamines on female oviposition.** Total number of eggs laid by *C. chinensis* females of strains **(A)** jC-S, **(B)** isC, **(C)** akC02, and **(D)** mC after injection with dopamine, octopamine, tyramine, serotonin, or Milli-Q water (control) over 5 days after injection. The number of eggs laid differed significantly among monoamine treatments on days 1 to 5 in jC-S, isC, and mC, and on days 1 and 5 (but not on days 2 to 4) in akC02 (Additional file 1: Table S2). Bars labeled with an asterisk differ significantly from the control within a strain (*P* < 0.05, Dunnett’s test). The numbers in parentheses show the sample size.

Repeated-measures two-way analysis of variance (ANOVA) revealed that all between-subject sources of variance (monoamine treatment, female strain, treatment × strain) and all within-subject sources of variance (days after injection, days × treatment, days × strain, days × treatment × strain) significantly affected the number of eggs laid (Table 2). Because of the significant interaction effects, separate one-way ANOVA tests were applied to the differences among monoamines in each strain and on each day (Additional file 1: Table S2). Relative to the control, tyramine significantly (*P* < 0.05) increased the number of eggs from day 1 and serotonin from day 4 in jC-S (Figure 2A) and mC (Figure 2D); octopamine did so from day 1 and tyramine on day 4 in isC (Figure 2B); and tyramine did so on day 1 in akC02 (Figure 2C). Results of monoamines on female receptivity and number of eggs laid by females are summarized in Table 3. Law dates of them were attached (Additional file 2).

**Table 2:** Results of repeated-measures two-way ANOVA of eggs laid by females of four strains in the monoamine injection experiment

**Source**	***df***	***F***	***P***
Between-subject			
Treatment	4	8.746	< 0.0001
Strain	3	15.593	< 0.0002
Treatment × strain	12	1.948	0.0274
Error	19	5.389	
Within-subject			
Day	1.92	4888.741	< 0.0001*
Day × Treatment	7.67	5.503	< 0.0001*
Day × strain	5.77	13.821	< 0.0001*
Day × treatment ×	23.06	2.140	0.0014*
Error	36.51	4.543	

*Corrected *P,* Greenhouse-Geisser ε = 0.4804.

**Table 3:** **Summary of effects of monoamines on reduction of female mating receptivity and eggs laid compared with control of each strain of** ***Callosobruchus chinensis***

**Trait**	**Strain**	**Dopamine**	**Octopamine**	**Tyramine**	**Serotonin**
Receptivity	jC-S		●	●	
	isC				
	akC02	●	●	●	●
	mC		●	●	
Egg laying	jC-S			● 1-5 d	● 4-5 d
	isC		● 1-5 d	● 4 d	
	akC02			● 1 d	
	mC			● 1-5 d	● 4-5 d

*“●”* indicates an effect; number indicate days on which effects on egg laying were found.

## Discussion

Artificial injection to *C. chinensis* strain jC-S females revealed a relationship between biogenic monoamines and female mating receptivity, oviposition, or both [43]. My results confirm that result and reveal differences among *C. chinensis* populations in the effects of monoamines on female receptivity and the number of eggs laid. In other studies, oral application of serotonin induced mate-rejection behavior in female *Pieris rapae* and increased the level of serotonin and its metabolites in the brains of females [38]; injection of tyramine reduced production of the sex pheromone bombykol in mated *Bombyx mori* females [39]; and octopamine and analogs applied to the dorsal surface of female *Plodia interpunctella* increased the number of eggs laid [40]. However, this is the first report to show differences among or within populations, which could reflect differences among genotypes, in the effect of monoamines on females of different strains of the same species.

Octopamine and tyramine significantly reduced female receptivity in jC-S and mC compared with the control (Figure 1). Females of both strains remated less frequently than those of the other strains (Additional file 1: Table S1). A previous study [41] showed that two kinds of male-derived substances reduced female receptivity: low-molecular-weight (MW) substances (<3 kDa), which act quickly (within 3–5 h and 1 day after injection), and higher-MW substances (>14 kDa), which act more slowly (2–4 days after injection). The jC-S and mC females responded more strongly than the other females to male substances that reduce female receptivity: jC-S females responded to the low-MW substances within 3 to 5 h and 1 day, and to the higher-MW substances by 2 and 4 days after injection, and mC females responded to the low-MW substances by 1 day and to the higher-MW substances at 2 and 4 days after injection [41]. In contrast, isC females, with a higher remating frequency (Additional file 1: Table S1), responded more weakly to male substances than the other strains: they did not respond to the low-MW substances, and responded to the higher-MW substances at 2 days after injection [41]. The receptivity of isC females was not reduced by the injection of any tested monoamine (Figure 1). Therefore, the effects of biogenic monoamines in these strains coincided with the degree of response to male substances. Likewise, akC02 females, which also respond more weakly than the other strains to male substances, did not respond to low-MW substances, and responded to high-MW substances at 2 and 4 days after injection [41]; however, they responded strongly to all four monoamines by reducing their receptivity (Figure 1). Female receptivity can also be reduced by other factors, such as mechanical stimulation during mating, insertion of the male’s aedeagus, mechanical pressure on stretch receptors in the female bursa, and the presence of sperm in the female spermatheca [21]. These factors may be more important than the composition of the seminal fluids in the reduced receptivity of akC02.

Tyramine significantly increased the number of eggs laid on days 1 to 5 and serotonin on days 4 and 5 by jC-S and mC (Figure 2A, D). Tyramine significantly increased the number laid on day 4 by isC females (Figure 2B) and on day 1 by akC02 females (Figure 2C). Octopamine significantly increased the number laid on days 1 to 5 by isC females (Figure 2B). Male-derived substances (>14 kDa) activate female oviposition, and females of jC-S and mC had stronger responses to them than females of isC and akC02; jC-S and mC females responded to these substances derived from males of each of the same four strains used here, but isC females did not respond to substances from three of the strains and akC02 females did not respond to any [42]. Thus, differences among the strains in the numbers of eggs laid coincided with the responses to male substances. The similarities and differences in the effects of the four monoamines, especially tyramine and serotonin, might be related to the effects of male substances.

Serotonin receptors have been identified in invertebrates [44]. Behavioral pharmacology and genetic studies of insect serotonin receptors revealed the specific involvement of receptor subtypes in modulating behaviors [45]–[48]. Furthermore, because *C. elegans* mutants of serotonin receptor genes *SER-1* and *SER-2* stopped laying in response to exogenous serotonin, and expression of *SER-1* in vulval muscles recovered the response to serotonin, serotonin acts on egg laying via the SER-1 receptor-coupled signaling pathway in vulval contraction [33]. Thus, the coincidences between the difference of the effects of serotonin on oviposition and the seminal fluid substances that stimulate oviposition among populations indicate that serotonin receptors are involved in responses to male substances that induce female oviposition in *C. chinensis*. Moreover, tyramine acts as an intermediate precursor for octopamine biosynthesis, but it also has diverse direct roles in physiology and behavior [28],[29]. It is possible that octopamine converted from the injected tyramine affected female mating behavior suggested by previous study [43]. Therefore, similarities and differences in male substances related to increased oviposition, and differences among strains, may be related to similarities and differences among the genes involved in the biosynthesis pathway from tyrosine via tyramine to octopamine, which is mediated by the enzymes tyrosine decarboxylase and tyramine-β-hydroxylase [30]–[32]. They may also be related to differences in the receptors for octopamine, tyramine, or both [28],[29], as isC females increased oviposition significantly after injection with octopamine (Figure 2B).

Females of the red flour beetle, *Tribolium castaneum*, that mated with males of the same genotype remated earlier [26] and their lifetime fecundity was lower [25] than females that mated with males of different genotypes, and females of *Musca domestica* that mated with males of the same genotype oviposited less than females that mated with males of different genotypes [23]. These studies suggest that females resist male manipulations that reduce their receptivity and induce their fecundity following mating, and that antagonistic coevolution occurs between the sexes as a result of female choice. In contrast, *Callosobruchus maculatus* females that mated with males of different genotypes remated earlier and their fecundity was lower than females that mated with males of the same genotype [24]. This indicates that coevolution between the sexes resulted from female resistance to male manipulations that reduce their receptivity and induce fecundity.

In addition to female responses, there were differences in the effects of these substances between male genotypes: jC-S male substances more strongly reduced female receptivity and activated oviposition [41],[42]. As mentioned above, jC-S females had stronger responses to male substances. Therefore, these results suggest that the ability of males to reduce receptivity and induce fecundity is selected for by the female, and that coevolution may have occurred between the sexes. These male manipulations appear to disadvantage the females—for example, male-derived substances reduce female longevity [42]—but might select for males with a strong ability to secure their paternity by enhancing their male offspring’s fitness, or might select for abilities linked to other traits that enhance benefits to females, such as nutrient availability or offspring viability [14],[17].

## Conclusion

The four biogenic monoamines produced different responses in the four female strains of *C. chinensis*. The differences in responses (reduced receptivity or increased oviposition) suggest the result of mutations in the biosynthesis pathways or the receptors for these monoamines. That is, choice among traits is mediated via such mutations. By examining variations between populations in the monoamine composition of male ejaculate, it should be possible to reveal details of the physiological mechanisms that underlie the differences among the female genotypes in their responses to differences in male ejaculate. These differences may lead to sexual selection as a result of female choice and the evolution of the substances in male seminal fluids as a result of coevolution between the sexes.

### Ethics

This study had been conducted under the ARRIVE guidelines, which are intended to improve the reporting of animal research on vertebrates or any regulated invertebrates.

## Methods

### Insects

The four strains of *C. chinensis* were maintained in a growth chamber at 25°C, 60% relative humidity, and a light–dark cycle of 16 L:8 D. Strains jC-S and mC were reared at the University of Tokyo [49],[50] and strains isC and akC02 at Okayama University [51],[52]. Approximately 100 adults of each strain were kept in separate plastic Petri dishes (height, 1.5 cm; diameter, 9.1 cm) at Okayama University. In April 2011, the beetles were transferred to a chamber with the same conditions at the National Agricultural Research Center, Tsukuba, Japan. All beetles were reared from eggs laid by randomly collected females on adzuki bean (*Vigna angularis* ‘Dainagon’) seeds (restricted to one egg per seed). Each seed was then transferred into a separate well of a 48-well tissue culture plate (Greiner Bio-One, Frickenhausen, Germany) and kept within the chamber described above. Beetles eclose within the bean and then emerge from it. Thus, virgin male and female adults were collected from the beans in the wells. Emerging females have mature eggs in their oviducts and bursa copulatrix [43].

### Injection of monoamines

Dopamine, octopamine (Nacalai Tesque, Kyoto, Japan), tyramine, or serotonin (Sigma-Aldrich, Tokyo, Japan) was dissolved in Milli-Q water to a final concentration of 10% (dopamine and octopamine, 0.53 M; tyramine, 0.58 M) or 2% (serotonin, 0.05 M). Serotonin does not dissolve well in water, so it was not possible to create a concentration greater than 2%.

Virgin females aged 1 to 4 days old were chilled on ice for a few minutes and fixed to agarose medium at room temperature by using fine forceps. Under a microscope, a hole was made between the second and fifth segments of the ventral abdomen with the forceps, and 0.05 μl of a monoamine solution or Milli-Q water (control) was injected through an ultra-fine glass capillary connected to an oil-pressure injector (Nanoject Auto-Nanoliter Injector, Drummond Scientific Company, Broomall, PA, USA).

At 3 to 5 h after the injection, the female and a virgin male of strain jC-S were placed in a small plastic Petri dish (height, 1.5 cm; diameter, 3.0 cm) in the chamber described above. The pair was then observed for 1 h to see whether the female mated.

To count the number of eggs laid, I immediately transferred the mated females into separate wells of a 24-well tissue culture plate (Nalge Nunc International K.K., Tokyo, Japan) and supplied one adzuki bean per female. I counted the eggs oviposited on each bean every day for 5 days. Data for females that died during that period were excluded from analysis. If eggs were laid on a bean, the bean was replaced with a new one.

### Statistical design

The effects of the monoamines on female mating receptivity were evaluated with a generalized linear model (GLM), using a binomial error distribution and a log link function, with mating (yes or no) as the response variable, and treatment (four monoamines and a control) and strain (four strains) as the independent variables. When the GLM revealed a significant interaction between strain and treatment, after significant effects were detected among treatments by the GLM with binomial errors, the method of Benjamini and Hochberg with a false discovery rate [53] was applied at the 5% significance level.

Cumulative total numbers of eggs (transformed as log[n + 1]) were compared using repeated-measures two-way ANOVA with “treatment” and “strain” as the between-subject factors and “days after injection” as the within-subject factor. Because Mauchly’s test of sphericity indicated a significant violation of the assumption of sphericity (*P* < 0.0001), significance levels for within-subject effects were calculated using a Greenhouse–Geisser correction of the degrees of freedom [54]. When a significant interaction effect was encountered among between-subject or within-subject sources of variance (Table 2), separate one-way ANOVAs were applied to the differences among strains. To compare variables between control and monoamines, when significant effects were detected in the separate one-way ANOVA, Dunnett’s test was used for multiple comparisons at the 5% significance level. All statistical analyses were performed in JMP v. 11.0.0 software [55].

## Abbreviations

5-HT: Serotonin

GLM: Generalized linear model

ANOVA: Analysis of variance

MW: Molecular-weight

## Competing interests

The author has no financial or non-financial competing interests.

## Additional files

## Supplementary Material

Additional file 1: Table S1. Rearing history and female remating frequency of each strain of *Callosobruchus chinensis.***Table S2.** Results of one-way ANOVAs testing the effects of monoamine treatment on the number of eggs laid by females in each strain at 1 to 5 days after injection. Click here for file

Additional file 2: **Date of mating receptivity.** Date of number of eggs laid by females. Click here for file
