# Supplementary material for: Genetic variation in the effect of monoamines on female mating receptivity and oviposition in the adzuki bean beetle, Callosobruchus chinensis (Coleoptera: Bruchidae)
Source: BMC Evol Biol. 2014 Aug 6;14:172. doi: 10.1186/s12862-014-0172-5 (PMC4360256; doi:10.1186/s12862-014-0172-5)
Supplement: Supplementary file 1 [file s12862-014-0172-5-S1.doc]

Table S1. Rearing history and female remating frequency of each strain of *Callosobruchus chinensis*

Strain	Collection year	Locality of population (latitude, longitude)	Number of founder adults	% Female remating[a]
jC-S	1936	Kyoto, Kyoto (34° 59' N, 135° 46' E)	No information	8.8
isC	1997	Ishigaki, Okinawa (24° 22' N, 124° 10' E)	> 200	38.4
akC02	2002	Sanyo, Okayama (34° 45' N, 134° 01' E)	> 50	29.8
mC	1960s	Morioka, Iwate (39° 42' N, 146° 11' E)	No information	5.9

[a]The data are the cumulative frequency of female remating for five days after first mating [45]

Table S2. Results of one-way ANOVAs testing the effects of monoamine treatment on the number of eggs laid by females in each strain at 1 to 5 days after injection.

Day	Strain	*df*	SS	*F*	*P*
1	jC-S	4	0.893	4.930	0.0013
	isC	4	1.469	2.820	0.0276
	akC02	4	0.737	2.526	0.0445
	mC	4	0.709	3.509	0.0095
2	jC-S	4	1.421	3.203	0.0171
	isC	4	3.043	3.752	0.0063
	akC02	4	0.870	2.126	0.082
	mC	4	1.875	4.399	0.0023
3	jC-S	4	1.981	3.544	0.0102
	isC	4	2.893	3.044	0.0194
	akC02	4	0.909	1.925	0.1109
	mC	4	2.525	3.858	0.0055
4	jC-S	4	3.085	5.003	0.0012
	isC	4	2.924	3.315	0.0127
	akC02	4	1.190	2.260	0.0669
	mC	4	3.340	4.537	0.0019
5	jC-S	4	4.035	6.606	< 0.0001
	isC	4	2.451	2.852	0.0263
	akC02	4	1.681	2.912	0.0245
	mC	4	3.348	4.406	0.0023
